# The Role of Home-Based Exercise in the Management of Peripheral Artery Disease

**DOI:** 10.1007/s11936-026-01143-4

**Published:** 2026-04-27

**Authors:** Carlos A. Ortega, Aaron W. Aday, Tara A. Holder, Brian R. Lindman, Alexander E. Sullivan

**Affiliations:** 1 Department of Medicine, Vanderbilt University Medical Center, Nashville, TN, USA; 2 Division of Cardiovascular Medicine, Department of Medicine, Vanderbilt University Medical Center, Nashville, TN, USA; 3 Vanderbilt Translational and Clinical Cardiovascular Research Center, Division of Cardiovascular Medicine, Department of Medicine, Vanderbilt University Medical Center, Nashville, TN, USA; 4 Department of Medicine, Division of Cardiology, Prisma Health-Upstate, Greenville, SC, USA; 5 Structural Heart and Valve Center, Division of Cardiovascular Medicine, Department of Medicine, Vanderbilt University Medical Center, Nashville, TN, USA

**Keywords:** Peripheral arterial disease, Claudication, Home-based exercise therapy

## Abstract

**Purpose of Review:**

Supervised-exercise therapy (SET) is a cornerstone intervention for management of peripheral artery disease (PAD) and is associated with improvement in ambulatory functional status. Despite strong evidence supporting the benefits of SET, accessibility and adherence remains a challenge, underling its full clinical potential. Home-based exercise therapy (HBET) has emerged as more accessible alternative to SET. This paper reviews the benefits of exercise in PAD, explores the role of home-based exercise therapy in the management of PAD, and describes the essential components of HBET in contemporary clinical practice.

**Recent Findings:**

HBET programs have demonstrated similar improvements in ambulatory function, including walking distance and pain-free walking distance, as established SET regimens. Recent clinical trials and meta-analysis have established HBET as a reasonable alternative to SET, as reflected in the latest multi-societal clinical guidelines.

**Summary:**

Functional and quality of life improvements with HBET parallel those with SET and outperform routine exercise education. Successful HBET should include both monitoring and behavioral intervention components to improve adherence and long-term symptomatic improvements. Providers should tailor HBET to the individual patient’s needs and limitations. Ongoing research aims to understand the optimal platform to deliver HBET programs to a wide range of patients with symptomatic PAD.

## Introduction

Peripheral artery disease (PAD) is an atherothrombotic disease of the lower extremities that affects more than 230 million people worldwide [1]. Patients with PAD may be asymptomatic, while others experience exertional leg symptoms or develop chronic limb-threatening ischemia, characterized by tissue lose and rest pain [2]. Even in asymptomatic patients with PAD there is often impaired ambulatory function [3–6]. The classical symptom of chronic symptomatic PAD is claudication. Those with typical claudication may experience lower extremity muscle fatigue, cramping, aching, or pain, that is reproducible with exertion and relieved by rest [2, 7]. Many patients however, especially women and those with diabetes or comorbid neuropathy, may not report typical claudication symptoms. These patients may report exertional tightness, numbness, fatigue, or cramping [2, 7, 8]. Claudication and other exertional leg symptoms lead to reduced mobility, diminished quality of life, and increased risk of major adverse cardiovascular events (MACE), including myocardial infarction, stroke, and cardiovascular-related death [9–11].

PAD management is a multi-dimensional and multi-disciplinary endeavor that includes medical management, exercise therapy, and revascularization therapies. Non-invasive medical and exercise therapies seek to reduce the risk of MACE and adverse limb outcomes, including revascularization and amputation, while simultaneously improving functional capacity [2, 12]. This includes optimization of modifiable risk factors such as hyperlipidemia, hypertension, diabetes, and tobacco use as well as antithrombotic pharmacotherapy tailored to the patient’s unique thrombotic and bleeding risk profile [2,3,12]. Supervised exercise therapy (SET) and revascularization procedures have both been shown to significantly improve walking distance and patient-reported outcome measures beyond that achieved by optimal medical therapy alone [13–15]. Since walking-based exercise program do not carry the same risks as invasive procedures, these are often first-line interventions for patients with chronic symptomatic PAD [2, 3, 12].

## Exercise Strategies in PAD

The Centers for Medicare and Medicaid Services (CMS) implemented reimbursement for SET in 2017 [16]. These programs consist of three, 30- to 60-minute sessions of therapeutic exercise each week for a total of 12 weeks. Sessions take place in a clinic, hospital, or cardiac rehabilitation center where exercise physiologist or nurse implement an exercise regimen under the direct supervision of a physician or advanced practice provider. Walking is the optimal exercise modality to maximize function improvements in patients with PAD, although arm and leg ergometry as well as resistance training are alternative modalities in those patients unable to walk [16, 17]. In light of this growing evidence base, multiple clinical trials have demonstrated durable improvements in functional status and quality of life with SET, similar to that of revascularization [13, 14].

In contrast to SET, the components of a home-based exercise therapy (HBET) program are not well defined (Table 1). Walking is consistently recommended as the first line form of exercise in HBET programs with other forms of exercise reserved for those with mobility issues [12]. The locations were the exercise takes place is flexible and determined by the patient. The frequency of exercise and duration of an HBET is recommended to be at least three times per week for at least 12 weeks [3, 6, 18]. There is flexibility in the length of exercise sessions with some recommending starting with 10 min sessions based on patient factors with a progressive increase in the duration of sessions [6]. The recommend length of exercise sessions ranges from 30 to 60 min [3, 6, 18]. Goal setting and tracking progress is recommended as part of HBET along with accountability [6].

## Benefits of Exercise Therapy for Chronic Symptomatic PAD

In chronic symptomatic PAD, the atherosclerotic obstruction leads to exercise-induced ischemia followed by rest-induced reperfusion [19, 20]. This process leads to multiple complex pathologic changes affecting the vasculature and skeletal muscles of the lower extremities [5, 6, 19, 20]. While it may appear paradoxical that exercise confers benefits in patients with PAD, high-intensity walking exercise that elicits claudication symptoms significantly improves 6-minute walk test (6MWT) distance and quality of life[21]. These benefits, however, are not seen with less intensive exercise, highlighting the importance of training into and beyond the onset of ischemia.

Recurrent ischemia in patients with PAD has been shown to increase inflammation, oxidative stress, altered metabolism, and capillary dropout [22–28]. These changes contribute to loss of skeletal muscle mass as well as increased fatty infiltration and fibrosis, which creates a self-perpetuating cycle of ischemic injury and maladaptive structural changes that contribute to mobility los [22, 29, 30]. In contrast, exercise therapy reduces epithelial-derived inflammation, increases oxygen uptake, promotes microvascular growth, and improves lower extremity strength and endurance [20, 31–35].

While the exact mechanisms by which high-intensity exercise promotes beneficial vascular and skeletal muscle remodeling are incompletely understood, both supervised and home-based exercise programs have been shown to improve walking distance and patient reported quality of life measure [36–41]. This lead to a Class 1 A recommendation for structured exercise, including SET and community-based (including structured home-based) programs in the most recent 2024 American College of Cardiology (ACC)/American Heart Association (AHA) Guidelines for the Management of Lower Extremity Peripheral Artery Disease [2].

## Limitations of Supervised Exercise Therapy in PAD

**Fig. 1:**
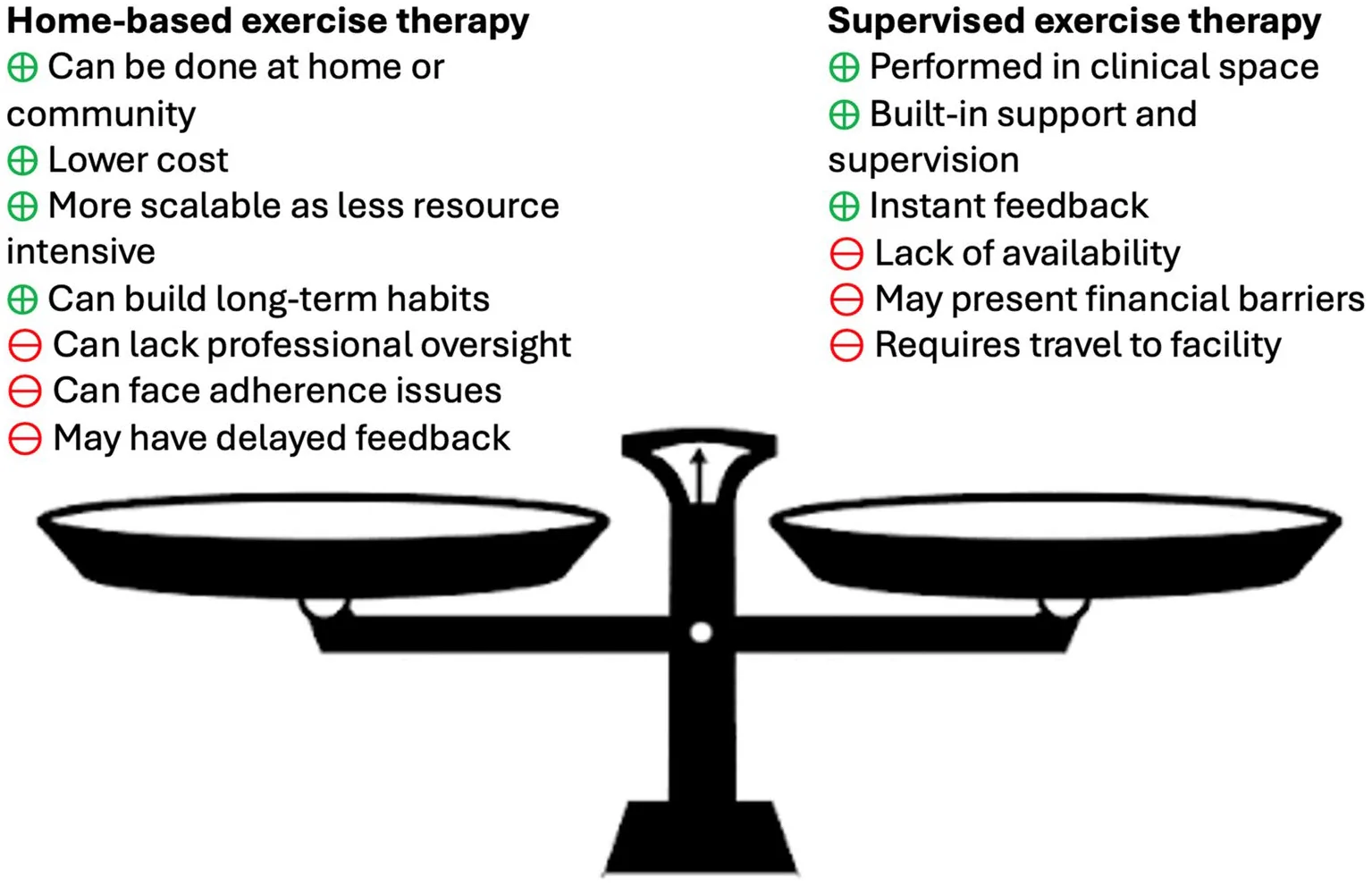
Comparison of HBET versus SET

Despite this evidence and reimbursement by CMS, SET remains underutilized [2, 8, 40–44]. A recent study of Medicare beneficiaries with claudication found that only 1.8% of those eligible for SET were enrolled, compared to 17.8% who were referred for revascularization [45]. Access is a significant barrier; up to 54% of vascular specialists report a lack of access to an exercise facility and 49% have never referred a patient for SET [42]. Even amongst those who are referred, only 10% complete the 12-week program [46]. Patient interest is an additional barrier with only 30–60% of patients being interested in participating in SET – citing cost and time consumption related to traveling three times per week to visits [42, 46–48]. Others report financial barriers that prohibit the copays for the 36 sessions [46]. Given the benefits to exercise therapy for patients with chronic symptomatic PAD but limitations in access and adherence, HBET has emerged as a potential alternative (Fig. 1).

## Advantages of HBET Programs and Potential Barriers

HBET offers flexibility by allowing individualized adjustment of session duration, frequency, and intensity. Unlike SET home-based exercise therapy, HBET does not require clinician oversight and can be performed in community or home settings. This approach provides several advantages, including the ability to exercise in a safe and familiar environment, which may reduce anxiety and enhance adherence, particularly for patients with mobility limitations or those living in areas with limited access to supervised programs. The reduced logistical burden, combined with the potential for remote monitoring or app-based support, may further improve accessibility and adherence among populations facing barriers to facility-based care. For uninsured or financially constrained patients, HBET also represents a more cost-effective alternative to SET [49].

Lack of walkable areas are an issue facing patients living in urban environments with studies demonstrating a correlation between urban planning and the amount of physical activity that patient’s perform [50, 51]. In these scenarios, HBET programs can be adapted to include non-walking forms of exercise, such as resistance training. The evidence for use of HBET in patients who use walking assistance devices is lacking and is not addressed in current guidelines [2, 12]. However, barring a contraindication to ambulation with a walking assistance device, such as significant gait instability, HBET can be adapted to individual walking abilities. Alternate forms of exercise such as resistance training are also effective and are reasonable alternatives included in clinical guidelines [2, 12, 52]. The ability to adjust the HBET regimen offers flexibility for each patient to tailor the regimen to their own limitations and goals.

## Behavioral Interventions and Monitoring in Effective HBET Programs

Effective home-based programs for PAD consistently incorporate goal setting, self-monitoring (such as exercise logs or wearable activity monitors), and regular accountability meetings with a coach or clinician, often via telephone or digital platforms [3, 17, 21, 53–63]. Programs that incorporate regular contact, feedback, and accountability mechanisms are equivalent in efficacy to supervised exercise programs, whereas those lacking these elements have been significantly less effective and durable [3, 6, 40, 64]. Behavioral support remains a cornerstone of successful HBET for PAD. Structured interventions that include group meetings, individualized counseling, and periodic feedback have been included in multiple studies that have found HBET to be superior to control groups [6].

While monitoring is an essential component of effective HBET, the type of monitoring has varied greatly in clinical trials. Most clinical trials have used phone calls as the method for monitoring progress and motivating participants to achieve their walking goals. Studies with more intensive monitoring, such as in-person sessions, home visits, and phone calls, have demonstrated improvement in the primary outcome of average daily steps in the HBET group compared to the control group [54, 55]. Studies with less intensive monitoring, such as the TrackPad Study—where the primary method of monitoring was a phone application with no in-person visits or phone calls— have also demonstrated improvement in the primary outcome of 6MWT in the HBET group compared the control group [62].

Physical activity monitors have been used in the majority of HBET clinical trials with pedometer, step activity monitors, and accelerometers being the most frequently used. Recently, a meta-analysis on tracking-based technologies demonstrated improvements in all walking ability parameters and in self-reported quality of life measures in those assigned to a tracking-based technology group compared to control groups [65]. For HBET programs, activity monitors facilitate self-monitoring, goal setting, and accountability, which are key behavioral strategies that improve adherence and clinical outcomes.

## Evidence for Home-Based Exercise Therapy for PAD

HBET for PAD has emerged as an increasingly important strategy for managing PAD given the limited access to and uptake of SET programs. Both the 2024 ACC/AHA/Multisociety Guideline for Management of Lower Extremity PAD and 2024 ESC Guidelines for the Management of Peripheral Arterial and Aortic Disease recommend consideration of HBET for patients with PAD [2, 12]. A review of the most relevant data supporting these recommendations can be found in Table 2.

HBET has consistently been demonstrated to be superior to various control groups. Multiple clinical trials have found an increase in peak walking time and an increase in claudication onset time in patients participating in HBET compared to usual care control groups [53, 59]. Other studies has found that HBET can increase the average daily steps taken by patients by over 1500 steps compared to usual care [54, 55]. Walking improvements with HBET are also durable, and have been shown to be superior to usual care in increasing 6MWT distance at 6 weeks, 3 months, 6 month, and 12 months in clinical trials [21, 56–58, 60, 62, 63]. Pooled data has demonstrated similar findings. A meta-analysis comparing HBET to walking advice found that HBET increased both maximal treadmill distance and pain-free treadmill walking distance by 210 m and 140 m, respectively [66]. Another meta-analysis by Golledge et al. found that HBET led to significant improvements in maximum walking distance, claudication onset time, physical activity time (measured by monitoring device), and 6MWT distance compared to controls [41].

Importantly, data supporting HBET have demonstrated some heterogeneity [67–69]. The HONOR trial compared HBET with telephone coaching and activity monitoring and did not find a difference between HBET and control group in terms of 6MWT distance at 9 month follow-up [69]. The authors concluded that a component of a successful HBET program includes periodic on-site visits. However, the TrackPAD study demonstrated a significant improvement in 6MWT distance with HBET versus usual care without in-person visits, utilizing a phone application as the primary mode of intervention mechanism for patients assigned to the HBET group [62].

Comparisons between HBET and SET have yielded inconsistent results and do not support HBET as a routine substitute for SET. Garner et al. (2011) found that HBET and SET significantly improved claudication onset time and peak walking time, but did not find a significant difference between the HBET and SET groups [53]. In the NEXT Step trial, HBET and SET were superior to resistance training in improving claudication onset time and peak walking time [59]. SET, however, resulted in a greater improvement than did HBET [59]. This has been supported by meta-analysis data which demonstrated greater improvement in treadmill distance and pain-free treadmill walking distance with SET compared to HBET [66]. After stratifying HBET into those that were monitored vs. unmonitored, monitored HBET produced similar walking improvements to SET, but unmonitored trials were inferior to SET [40].

## HBET Heterogeneity and Role in Contemporary Clinical Practice

While the most recent 2024 ACC/AHA Guidelines for the Management of PAD give a Class IA recommendation for HBET in patients with claudication, the 2024 ESC Guidelines are less optimistic, offering a Class 2a, Level A recommendation for HBET when SET is not available or feasible [2, 12]. This disagreement between the two societal guidelines highlights the need for a better understanding of the optimal components of HBET, as well as the significant heterogeneity in the study protocols and results of trials published to date. Comparative trials have consistently demonstrated superiority of SET to HBET. While recent meta-analysis data suggested that the walking benefits were greater with HBET than SET, the included trials did not compare SET and HBET in a head-to-head fashion [40, 70]. Many have hypothesized that supervision and external motivation are the components of SET that have been difficult to reliably reproduce in community-based program, and that only those patients who are independently motivated to comply and adhere to high-intensity exercise stand to benefit from these programs [71]. Successful HBET trials, such as GOALS and LITE, have all incorporated regular (at least 5x/week), high-intensity exercise with monitoring systems and behavioral interventions. Without the monitoring from a pedometer, accelerometer, or step log and regular coaching or cognitive therapy, long-term HBET is challenging. No monitoring and behavioral intervention has ever been compared to others, and so precise prescription is not feasible. Additionally, wearable activity monitors and regular exercise physiology visits are not necessarily available and/or financially accessible to all patients with claudication. Some have tried to overcome this using app-based programs, but ultimately there remain unanswered questions surrounding the most optimal and scalable HBET program and how best to delivery such a program to patients from a variety of socioeconomic backgrounds [72].

While HBET have low rates of attrition, studies have demonstrated that compliance to the prescribed walking program may wane in the absence of in-person touchpoints, especially after 4–6 weeks of exercise. Walking distance and step counts may similarly plateau at this point as patient motivation declines [71]. This is also seen in clinical trials, such as GOALS, that support the prescription of HBET. Patients randomized to HBET had greater walking improvement during the first 6 months when there were regular meetings and exercise sessions, but this improvement was less as touchpoints became remote, and monitoring frequency decreased [56–58].

Basic exercise advice alone has been shown to be insufficient for patients with PAD, as demonstrated by multiple clinical trials and meta-analyses [2, 21, 41, 53, 63, 66, 68]. For patients who are unable to participate in SET, HBET offers a practical and accessible alternative [2, 12].

## Recommendation for HBET Program

**Fig. 2:**
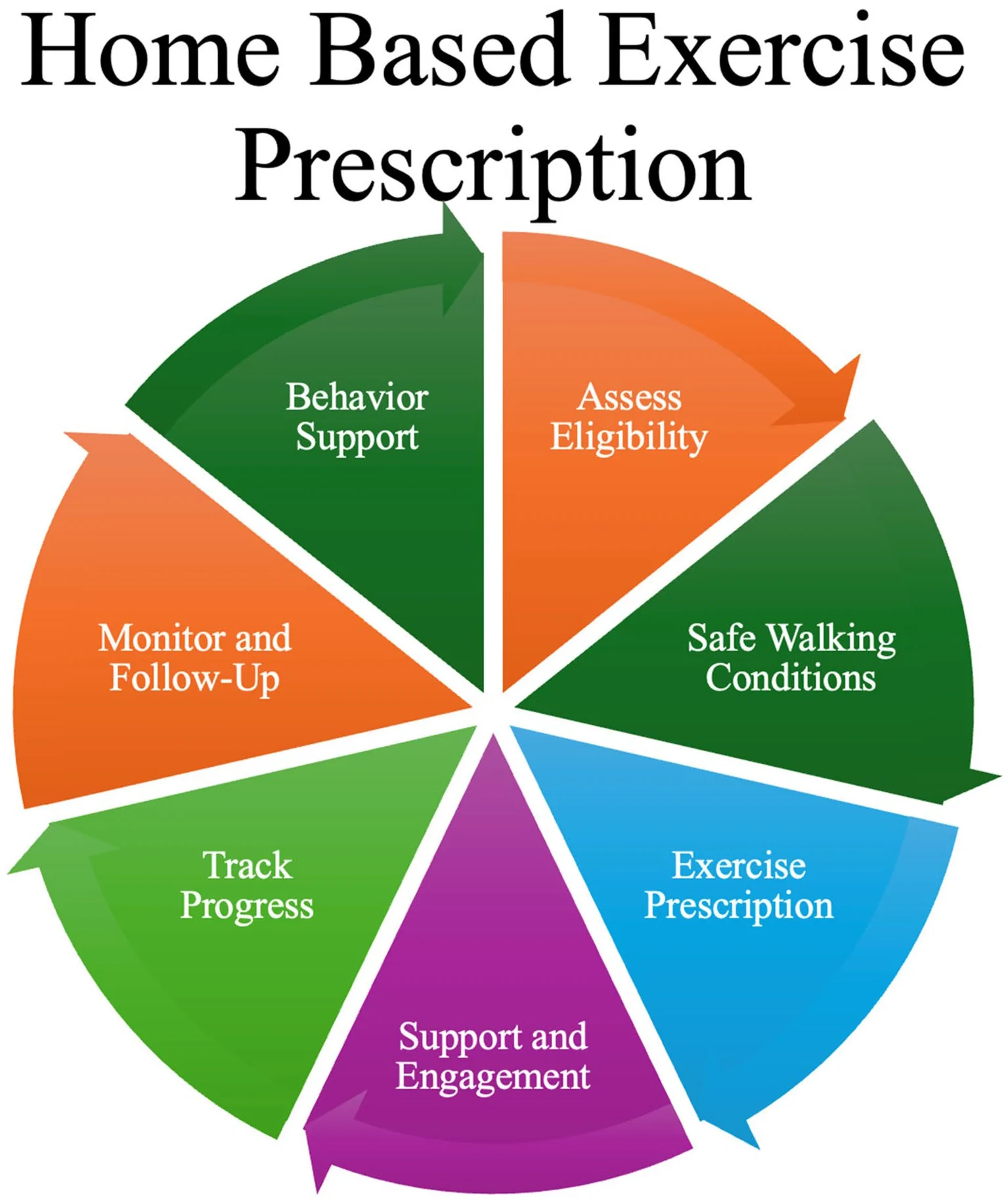
Components of a Successful Home-Based Exercise Program for the Vascular Clinician

In our current clinical practice, there are many patients who qualify for SET but are unable to attend due to a variety of barriers. For these patients, we recommend home- or community-based exercise and counsel them on the importance and potential benefits of exercise (Fig. 2). We first work with the patient to identify safe walking terrain, as many patients either do not feel safe walking in their neighborhood or live in an area with sidewalks or other flat walking structures. Patients are encouraged to wear comfortable, supportive shoes that will not result in recurrent trauma that might precipitate blister or wound formation. As discussed, there is currently no established “best” walking program, and so we advise the patient to aim for 3–5, 30-minute walking sessions per week. We emphasize the need to walk until claudication symptoms begin and then to continue to walk for an additional 30–60 s as a benchmark to reach “high-intensity” exercise. Spousal or family engagement, especially if present during the clinic visit, promotes socialization during exercise and adherence. For patients with access to smart technology or pedometric, we ask them to log their steps to assess improvement over time. Patients otherwise can monitor walking times, especially if they walk on the same track or path each time, to assess for improvement. Alternatively, they can track the time it takes them to develop claudication and strive to walk beyond that time. Routine check-ins can be challenging, but we monitor progress at least every 3–6 months with a telehealth visit, which is also an opportunity to continue medication and risk factor optimization.

Exercise management often parallels smoking cessation; many patients require several attempts before finding consistency and so counseling at each visit is important. Community can help with accountability, and so finding common goals with friends and family or walking groups is also helpful. While HBET requires vigilance of both the patient and provider, it can contribute significantly to the functionality and quality of life in patients with PAD.

## Future Directions

Several ongoing trials aim to evaluate delivery methods of HBET. The Telehealth Delivered Home-based Walking for Vets With Peripheral Artery Disease (TREK-PAD) trial is actively enrolling with in the VA system [73]. This study utilizes a walking program that includes information on healthy walking and motivational messages delivered via web-based vs. telehealth platform. Another randomized trial, Gamification-Augmented Home-Based Exercise for Peripheral Artery Disease (GAMEPAD), will compare a gamification-enhanced home-based walking program to conventional HBET. Unlike prior HBET trials which utilized a control of usual care or a “go out and walk” strategy, both arms will receive step-monitoring devices and regular coaching to understand the potential benefits of a gamified interface [74]. Finally, as smartphone technology becomes more widely available, multiple trials are utilizing novel app-based platforms to deliver walking programs and behavioral interventions to patients conveniently and in a manner that can be tailored to fit their lifestyle [75].

## Conclusions

Patients with PAD are at risk for significant morbidity and mortality. Exercise is a cornerstone therapy to mitigate this risk and improve long-term outcomes. While SET remains the standard-of-care, HBET is a feasible and effective option for patients who cannot attend SET due to access, financial, or logistical barriers. There is no widely available HBET program, and clinicians should provide patients with claudication a “HBET prescription.” This should include specific instructions on walking location, monitoring metrics, and clinical touchpoints. Progress and adherence should be closely monitored with frequent programmatic education and modification if needed.

## Figures and Tables

**Table 1:** Comparison of the Components of Supervised Exercise Therapy Programs and Home-Based Exercise Therapy Programs

Component	Supervised exercise therapy program	Home-based exercise therapy program
Location	Hospital or outpatient facility	Home, community, or neighborhood
Supervision	Direct, by qualified health care professional	Self-directed, +/− professional guidance and counseling
Exercise Modality	Treadmill walking (primary); alternatives as needed	Walking-based (primary); alternatives as needed
Session Frequency & Duration	3 times/week, 30–60 min per session, 12 weeks in duration	Variable but similar in frequency/duration to supervised exercise therapy
Progression	Incremental increases in walking time and intensity	Incremental increases in walking time, distance, and speed as tolerated
Behavioral Support	Provided during in-person sessions	Behavioral change techniques (coaching, activity monitors) recommended
Monitoring	Continuous, in-person	May include periodic check-ins which can be virtual or in-person
Insurance Coverage	Covered by Medicare, commercial	N/A
Co-Pay	May be required	N/A

**Table 2:** Summary of Clinical Evidence for Home-Based Exercise Therapy in PAD

Study name (Author, year)	Study size and groups	Frequency, duration (weeks), and form of HBET exercise	HBET behavioral intervention	Activity monitor	Primary Outcome results	Notable Secondary outcomes results
Studies that Support Routine Prescription of Home-Based Exercise Programs						
Gardner, 2011^53^	119 • HBET: 40 • SET: 40 • Usual care control group: 39	• 3x/week for 12 weeks • Walk to near maximal claudication pain	Review of step activity monitor data and logbook for 15-minutes with exercise physiologist who provided feedback during weeks 1, 2, 4, 6, 8, 10, and 12	Step activity monitor	Claudication onset time (s) • Change at 3-months: 134 in HBET vs. 165 in SET vs. −16 in control group*^a^ Peak walking time (s) • Change at 3-months: 124 in HBET vs. 215 in SET vs. −10 in control group*^a^	BASIC score • Change at 3-months: 1.4 in HBET vs. 0.6 in SET vs. −0.6 in control group*^a^ Maximum 60-minute cadence (strides/min) • Change at 3-months: 2.5 in HBET vs. 0.1 in SET vs. −1.3 in control group*^e^ Average cadence (strides/min) Change at 3-months: 1.1 in HBET vs. −0.1 in SET vs. −0.3 in control group*^c^
Cunningham, 2012^54^ Cunningham, 2013^55^	58 • HBET: 28 • Usual care control group plus researcher contact: 30	• 16 weeks, frequency not reported • Walking	Two 1-hour sessions one week apart delivered by trainee health psychologist using motivation interviewing techniques with emphasis on changing barrier and creating action plans and way to overcome barriers from following through on action plans. Total of 5 home visits during and two phone calls at weeks 6 and 12.	Pedometer	Walking behavior (steps) • 1575.63 more steps in the HBET group than in the control group*	
GOALS McDermott, 2013^57^ McDermott, 2014^56^ McDermott, 2015^58^	194 • HBET: 97 • Health education control group: 97	• 5x/week for 24 weeks • Walking to severe discomfort	Weekly meeting for 45 min with group faciliatory followed by 45-minute walking session on indoor track in the intervention group for 6 months followed by telephone calls biweekly in months 7 to 9 and once-monthly in months 10 to 12	Accelerometer	6MWT (m) • Change at 6-months: +42.4 in HBET vs. −11.4 in control group* • Change at 12-month: +26.5 in HBET vs. −7.6 in control group*	WIQ distance score: • 6-months: 47.4 in HBET vs. 34.4 in control* WIQ speed score: • 6-months: 47.7 m in HBET vs. 36.6 m in control* • 12-months: 46.5 m in HBET vs. 36.5 m in control* Short Physical Performance Battery: • 12-months: 10.33 in HBET vs. 9.81 in control*
NEXT Step Gardner, 2014^59^	180 • HBET: 60 • SET: 60 • Resistance training control group: 60	• 3x/week for 12 weeks • Walking to mild-to-moderate claudication	Review of step activity monitor data and logbook for 15-minutes with research staff who provided feedback during weeks 1,4, 8, 12	Step activity monitor	Claudication onset time (s) • Change at 3-months: 104 in HBET vs. 170 in SET vs. 7 in control group*^a^ Peak walking time (s) • Change at 3-months: 110 in HBET vs. 192 in SET vs. 22 in control group*^b^	6MWT (m) • Change at 3-months: 45 in HBET vs. 15 in SET vs. 4 m in control group*^c^ Time to minimum calf StO2 (s) • Change at 3-months: 146 in HBET vs. 142 in SET vs. 27 in control group*^a^ Recovery half-time of calf StO2 (s) • Change at 3-months: −76 in HBET vs. −71 in SET vs. −4 in control group*^a^ High-sensitivity c-reactive protein (mg/L) • Change at 3-months: −1.82 in HBET vs. −0.32 in SET vs. −0.59 in control group*^d^
Tew, 2015^60^	23 • HBET: 14 • Usual care control group: 9	• 7x/week for 6-weeks • Walk at pace that elicits strong ischemic leg symptoms	SEDRIC program; Three hour education session by trained educators on the SEDRIC program prior to starting HBET followed by support in setting step goals and a telephone call to discuss goal setting, review progress, and support maintenance of behavior change.	Accelerometer	6MWT (m) • Change at 6-weeks: +22.9 in HBET vs. −20.6 in control group*	WELCH score • Change at 6-weeks: +12.4 in HBET vs. −7.9 in control group* WIQ speed score • Change at 6-weeks: +8.7 in HBET vs. −3.6 in control group* WIQ distance score • Change at 6-weeks: +12.5 in HBET vs. −0.9 in control group* WIQ stair climbing score Change at 6-weeks: +12.5 in HBET vs. −0.9 in control group*
Duscha, 2018^61^	20 • HBET: 10 Usual care control group: 10	• 7x/week for 12 weeks • Walking to goal number of steps above baseline	Provided with personalized exercise prescription based on steps per day and study staff provided motivation and feedback during study period.	Wearable activity monitor	Peak VO2 (ml/kg/min) • Change at 3-months: +20.3% in HBET vs. +1.0% in control group* Claudication onset time (s) • Change at 3-months: +204.6 in HBET vs. −21.0 in control group*	Peak walking time (s) • Change at 3-months: +227.6 in HBET vs. + 22.4 in control group*
LITE Trial McDermott, 2021^21^	305 • High-intensity HBET: 120 • Low-intensity HBET: 120 Health education control group: 65	• 5x/week for 52 weeks • High-intensity group: walking at pace to elicit ischemic leg symptoms • Low-intensity group: walking at pace that does not elicit ischemic leg symptoms	Weekly visits with a coach in weeks 1–4 for the high- and low-intensity exercise groups followed by weekly telephone calls by coach to assist with adhering to the prescribed exercise	Accelerometer	6MWT (m) • Change at 12-months: +34.5 in high-intensity HBET vs. −6.4 in low-intensity HBET* • Change at 12-months: +34.5 in high-intensity HBET vs. −15.1 in control group* Change at 12-months: −6.4 in low-intensity HBET vs. −15.1 in control group^NS^	Maximal treadmill walking time (minutes) • Change at 12-months: +1.8 in high-intensity HBET vs. +0.7 in low-intensity HBET* • Change at 12-months: +1.8 in high-intensity HBET vs. +0.4 in control group* WIQ distance score: • Change at 12-months: +13.7 in high-intensity HBET vs. +1.1 in control group* • Change at 12-months: +14.6 in low-intensity HBET vs. +1.1 in control group* WIQ speed score: • Change at 12-months: +11.7 in high-intensity HBET vs. −5.0 in control group* Change at 12-months: +17.2 in low-intensity HBET vs. −5.0 in control group*
Track-PAD Study Paldan, 2021^62^	39 • HBET: 19 • Standard of care: 20	• NR, 12 weeks • Walking to ischemic leg symptoms	The phone app was designed to suggest weekly SET units goal based on prior performance, provided feedback after completion of a SET unit, recorded personal achievements, and provided a leaderboard.	Track-PAD phone application	6MWT (m) • Change at 3-months: +86.0 in HBET vs. −38.8 in control group*	-
The MOSAIC Randomized Clinical Trial Bearne, 2022^63^	190 • HBET: 95 • Usual care control group: 95	• 3x/week for 12 weeks • Walking at pace that elicits moderate ischemic leg symptoms	Physical therapists led two in-person 60 min session in weeks 1 and 2 followed by two 20-minute sessions in weeks 6 and 12	Pedometer	6MWT (m) • Change at 3-months: +22.3 in HBET vs. +9.2 in control group*	WELCH score • Change at 6-months: +6.6 in HBET vs. −1.4 in control group* B-IPQ score • Change at 6-months: −4.3 in HBET vs. +2.0 in control group* SR-MWD test (m) • Change at 3-months: +298 in HBET vs. +51 in control group*
Studies that *DO NOT* Support Routine Prescription of Home-Based Exercise Programs						
Collins, 2011^67^	145 • HBET: 72 • Attention control group: 73	• 3x/per week for 12 weeks • Walking at intensity that elicits moderate ischemic leg symptoms	Two 1-hour session with exercise instructor during baseline period, walking with exercise instructor in a group 1x/week for 12 weeks during study period, and bi-weekly phone calls.	Pedometer	Maximal walking distance on Gardner-Skinner graded exercise treadmill test (m) • Change at 6-months 39.2 in HBET vs. 24.5 in control group^NS^	Walking speed (units) • Change at 6-months: +5.7% in HBET vs. −1.9% in control group*
Mays, 2015^68^	20 • HBET: 10 • Usual care control group: 10	• 3x/week for 14 weeks • Walking at intensity that elicits moderate ischemic leg symptoms	Training, monitoring and coaching program; Initial in-hospital exercise training on treadmill 3x/week for 2 weeks followed by 12-weeks of walking in the community with monitoring via pedometer and coaching to provide guidance and address barriers.	Pedometer	Peak walking time (min) • Change at 14-weeks: +21.3% in HBET vs. 0% in control group^NS^	Claudication onset time (s) • Change at 14-weeks: +28.0% in HBET vs. −13.7% in control group*
*The HONOR Study* McDermott, 2018^69^	200 • HBET: 99 • Usual care control group: 101	• 5x/week for 36 weeks • Walking	Weekly meeting with a coach for 2 weeks followed by weekly meeting with a group and a coach for 2 weeks. Then structured telephone calls weekly for the first 2 months, every other week for the next 6 weeks, and once a month for the following 6 weeks	Wearable activity monitor	6MWT (m) • Change at 9-months: +5.5 in HBET vs. 14.4 in control group^NS^	WIQ distance score • Change at 9-months: 10.6 in HBET vs. 4.8 in control group^NS^ PROMIS pain interference: • Change at 9-months: 0.7 in HBET vs. −2.8 in control group*

* Indicates significant difference, p<0.05; NS: not significant difference;

a : SET significantly different than control, HBET significantly different than control, no significant difference between is HBET and SET;

b : SET significantly different than control, HBET significantly different than control, SET significantly different than HBET;

c : no significant difference between SET and control, HBET significantly different than control, SET significantly different than HBET;

d : no difference between groups, significant decrease from baseline in HBET group;

e : no significant difference between SET and control, HBET significantly different than control, no significant difference between is HBET and SET; NR: not reported.

## Data Availability

No datasets were generated or analyzed during the current study.

## Opinion Statement

Monitored home-based exercise therapy (HBET) is an effective substitute for supervised exercise therapy (SET) in patients with access, financial, or logistical barriers to center-based exercise. While these programs can produce similar functional improvements to SET, no standardized program has been commercialized. Successful HBET programs include a functional monitoring component (pedometer, accelerometer, etc.) and a behavioral intervention. In the absence of a ready-made HBET program, we recommend that clinicians provide patients with PAD a “HBET prescription,” which includes specific direction on walking location, monitoring metrics, and follow-up. Adherence and progress should be assessed regularly with programmatic modifications as appropriate.

## Abbreviations

B-IPQ: Brief Illness Perceptions Questionnaire score
BASIC: Baltimore Activity Scale for Intermittent Claudication
GOALS: Group Oriented Arterial Leg Study
HBET: home-based exercise therapy
HONOR: HOme-based moNitORed Exercise for PAD
LITE: Low InTensity Exercise Intervention in PAD
MOSAIC: Motivating Structured Walking Activity in Intermittent Claudication
PROMIS: Patient-Reported Outcomes Measurement Information System
SEDRIC: Structured EDucation for Rehabilitation in Intermittent Claudication
SET: Supervised Exercise Training
SR-MW: Self-Reported Maximum Walking Distance
StO2: Hemoglobin Oxygen Saturation
TrackPAD: Digital Support for Supervised Exercise Therapy in Peripheral Arterial Disease
WELCH: Walking Estimated Limitation Calculated by Histor
WIQ: Walking Impairment Questionnaire
6MWT: Six-Minute Walk Test
